# Translational analysis and final efficacy of the AVETUX trial – Avelumab, cetuximab and FOLFOX in metastatic colorectal cancer

**DOI:** 10.3389/fonc.2022.993611

**Published:** 2022-12-20

**Authors:** Joseph Tintelnot, Inka Ristow, Markus Sauer, Donjete Simnica, Christoph Schultheiß, Rebekka Scholz, Eray Goekkurt, Lisa von Wenserski, Edith Willscher, Lisa Paschold, Sylvie Lorenzen, Jorge Riera-Knorrenschild, Reinhard Depenbusch, Thomas J. Ettrich, Steffen Dörfel, Salah-Eddin Al-Batran, Meinolf Karthaus, Uwe Pelzer, Axel Hinke, Marcus Bauer, Chiara Massa, Barbara Seliger, Claudia Wickenhauser, Carsten Bokemeyer, Susanna Hegewisch-Becker, Mascha Binder, Alexander Stein

**Affiliations:** ^1^Department of Oncology and Hematology, Bone Marrow Transplantation with Section Pneumology, University Medical Center Hamburg-Eppendorf, Hamburg, Germany; ^2^Department of Diagnostic and Interventional Radiology and Nuclear Medicine, University Medical Center Hamburg-Eppendorf, Hamburg, Germany; ^3^Department of Internal Medicine IV – Oncology/Hematology, Martin-Luther-Universitat Halle-Wittenberg, Halle, Sachsen-Anhalt, Germany; ^4^Hämatologisch-Onkologische Praxis Eppendorf, Hamburg, Germany; ^5^Department of Internal Medicine III (Haematology/Medical Oncology), Technical University of Munich Hospital Rechts der Isar, Munchen, Bayern, Germany; ^6^University Hospital of Giessen and Marburg, Marburg, Hessen, Germany; ^7^Private Practice Onkodoc GmbH Götersloh, Götersloh, Nordrhein-Westfalen, Germany; ^8^Department of Internal Medicine I, University Hospital Ulm, Ulm, Baden-Wörttemberg, Germany; ^9^Private Practice Onkozentrum Dresden, Dresden, Sachsen, Germany; ^10^Institute of Clinical Cancer Research Institut für Klinisch-Onkologische Forschung (IKF) at Northwest Hospital, Frankfurt, Hessen, Germany; ^11^Department of Hematology and Oncology, Munich Hospital Neuperlach, Munchen, Bayern, Germany; ^12^Department of Hematology, Oncology and Tumorimmunology, Charite Universitatsmedizin Berlin, Berlin, Germany; ^13^Clinical Cancer Research Consulting (CCRC), Dösseldorf, Germany; ^14^Institute of Pathology, Martin Luther University Halle Wittenberg, Halle, Sachsen-Anhalt, Germany; ^15^Institute of Medical Immunology, Martin-Luther-Universität Halle-Wittenberg, Halle, Germany; ^16^Institute of Pathology, University Hospital Halle, Halle, Germany

**Keywords:** deepness of response, ETS, T cell diversity, MSS mCRC, immunotherapy

## Abstract

**Introduction:**

In metastatic colorectal cancer (mCRC), the efficacy of immune checkpoint blockade (ICB) has so far been limited to patients with microsatellite instability high tumors (MSI-H). Unfortunately, most mCRC patients suffer from non-immunogenic microsatellite stable (MSS) tumors. Therefore, new combinatorial strategies are urgently needed to enhance the immunogenicity of MSS tumors to finally increase the number of patients benefiting from ICB.

**Methods:**

The AVETUX trial aimed to combine the PD-L1 antibody avelumab with the standard of care chemotherapy combination FOLFOX and the anti-EGFR antibody cetuximab. Furthermore, we performed a central radiological review of the pre- and on-treatment computed tomography scans to better define the individual response to treatment.

**Results and Discussion:**

In total, 43 patients were treated of which 39 patients were confirmed as RAS/BRAF wildtype in central tissue review and finally response evaluated. A final progression-free survival (PFS) of 11.1 (range: 0.8 to 22.3 months) and a herein updated final overall survival (OS) of 32.9 months (range: 0.8 to 47.1 months) was reached. We observed a strong median depth of response of 67.5% tumor shrinkage and deepness of response correlated significantly with survival. On the other hand, early tumor shrinkage was not an indicator of better outcome at a cut-off of 20% (median values). In a next step, we correlated the individual best radiological response with potential ICB response biomarkers and found that the clonality and diversity, but not frequency of tumor infiltrating lymphocytes (TiLs) and peripheral blood mononuclear cells (PBMCs), strongly correlated with response. In summary, we report the final overall survival of the AVETUX trial and propose T cell clonality and diversity as a potential marker to predict response to chemo-immunotherapy combinations in MSS mCRC by performing a central radiological review.

**Clinical Trial Registration:**

ClinicalTrials.gov, identifier (NCT03174405).

## 1 Introduction

Most tumors of patients suffering from metastatic colorectal cancer (mCRC) are microsatellite stable (MSS) tumors (1). However, the efficacy of immunotherapies such as immune checkpoint blockade (ICB; PD-1/PD-L1 inhibition with or without CTLA4 inhibitors) has so far been limited to patients with high microsatellite instability (MSI-H) among mCRC patients (2–6). Given the success of immunotherapies in MSI-H mCRC or other cancer entities, several approaches have been or are being investigated to increase the efficacy of ICB in MSS mCRC patients (7–10). In MSS mCRC, the tumor microenvironment is composed of immunosuppressive myeloid-derived suppressor cells or regulatory T cells that prevent cytotoxic T cells to exploit their tumor reactive potential. The chemotherapeutic 5-FU can eliminate immunosuppressive cells from the tumor microenvironment, allowing an anti-tumor immune response to emerge (11). Furthermore, oxaliplatin and the anti-EGFR antibody cetuximab can induce immunogenic cell death in cancer cells that directly lead to activation of tumor reactive T cells and expression of the co-inhibitory receptors PD-L1 and LAG3 (12, 13). Thus, the AVETUX trial aimed to analyze the combination of FOLFOX (5-FU, folinic acid and oxaliplatin) and cetuximab with the anti-PD-L1 antibody avelumab in 43 first line mCRC patients. Overall, a reported median PFS of 11.1 did not suggest a strong benefit for all treated patients, but encouraging rates of secondary resection and the emergence of anti-avelumab escape variants suggest a benefit for subpopulations (14). To identify subgroups of patients who might benefit from additional ICB treatment to standard of care chemotherapy and anti-EGFR treatment, we first performed a central radiological review to assess each patient’s response more accurately. In a next step, we correlated the observed response with validated markers of response to single-agent immunotherapy treatment.

## 2 Methods

### 2.1 Clinical trial

The main inclusion criteria were: *RAS/BRAF* wild-type mCRC, ≥18 years of age; Eastern Cooperative Oncology Group (ECOG) performance status 0 or 1; no prior chemotherapy for metastatic disease (adjuvant chemotherapy allowed if completed more than 6 months before study entry); at least one measurable disease lesion (based on Response Evaluation Criteria in Solid Tumors (RECIST 1.1) (15); adequate organ function. The trial was conducted at 10 centers in Germany in compliance with the Declaration of Helsinki. The study protocol was approved by the local ethics committees and authorized by the competent authority. All participants provided written informed consent. The trial is registered with ClinicalTrials.gov (NCT03174405). The AVETUX regimen included avelumab at a dose of 10mg/kg intravenously (IV) over 60 to 90 min (bi-weekly from cycle 2 onwards), cetuximab at a dose of 250 mg/m^2^ IV over 60 to 90 min (weekly, first dose 400mg/m^2^) and a modified FOLFOX6 with oxaliplatin at a dose of 85 mg/m2 IV (day 1), 5-FU 400 mg/m^2^ IV bolus (day 1) and 5-FU 2400 mg/m^2^ IV continuous infusion (day 1 to 2), and LV at a dose of 400 mg/m^2^ IV. Disease assessment was performed within 28 days before the first dose of study treatment and every 4 cycles (8 weeks) thereafter until 6 months, followed by assessments every 3 months thereafter. Avelumab was not added until the second cycle for clinical and translational reasons. First, to ensure tolerability of FOLFOX and cetuximab (and potential allergic reactions) before adding a fourth drug. Second, to have a control of immune activation associated with avelumab. The primary endpoint was progression-free survival rate at 12 months; secondary endpoints were overall response rate, early tumor shrinkage, progression free and overall survival, and toxicity. We calculated the number of included patients as follows: Efficacy assumptions were derived from historical data. The primary efficacy endpoint was PFS rate at 12 months (PFSR@12) based on the on-treatment population (ITT). In several randomized trials on the chemotherapy/cetuximab combination in RAS/BRAF wildtype MCRC, PFSR@12 is about 40% (16, 17). The probability of accepting the experimental therapy as promising (≥57% PFSR@12) in terms of efficacy, in spite of a true PFSR@12 of ≤40% only was set at 0.1 (type I error), whereas the probability of erroneously rejecting the experimental therapy as not sufficiently efficient (≤40%), although the true PFSR@12 is promising (≥57%) was set at 0.2 (type II error, corresponding to a power of 80%). Initially, 43 patients were recruited, but 4 had to be excluded due to central pathological analysis and observed RAS/BRAF mutation. In addition, 3 patients with dMMR status were excluded from the formal analysis (n=36 patients). Finally, CT scans of 3 patients could not be retrieved, so 33 patients were evaluated centrally.

The legal funder (sponsor) of the trial is the AIO Studien gGmbH, Berlin Germany. Merck KGaA Darmstadt, Germany, as part of an alliance between Merck KGaA and Pfizer supported the trial with study medication and a research grant to the AIO Studien gGmbH. Merck KGaA had no role in the design and conduct of the trial; collection, management, analysis, and interpretation of the data; or the decision to submit the manuscript for publication. Merck KGaA and Pfizer reviewed the final manuscript prior to journal submission.

### 2.2 Biomaterials

Peripheral blood of participants was collected in STRECK cell-free DNA BCT tubes at baseline and after 3 cycles of chemotherapy. In addition, formalin-fixed paraffin-embedded (FFPE) tissue was obtained after biopsy or surgical removal of the tumor before initiation of treatment.

### 2.3 Immunohistochemistry

Immunohistochemistry was performed for the following antibodies PD-L1 (PD-L1 antibody MA5-16841 from Thermo Fischer; IC score), CD56 (NK cells) and CD16 (Myeloid cells) as described previously (14). In brief, 3 µm thick sections of FFPE tissue were stained with the indicated antibodies and sections were imaged using an Olympus BX50/51 microscope, equipped with a Jenoptik PROGRES GRYPHAX ARKTUR camera.

### 2.4 T cell receptor repertoire sequencing

T cell receptors of blood and TiLs were analyzed by amplification of T cell receptor beta chain (TRB) followed by next-generation sequencing as previously described (14). T cell clonality was calculated and the Shannon diversity was measured as an indicator for T cell diversity as described earlier (14). Analyses were carried out using R (version 3.4.4) and the package tcR. The datasets generated from this study can be found in the European Nucleotide Archive (ENA, ID: PRJEB35507).

### 2.5 Response assessment according to RECIST 1.1 by central readers

Tumor burden at baseline and under therapy were assessed in consensus of two independent radiologists specialized on oncologic imaging according to the response evaluation criteria in solid tumors RECIST 1.1 (15) using the dedicated software solution Mint Lesion version 13 (Mint Medical Inc., Heidelberg, Germany). Readers were blinded to all clinical patient data during the evaluation. Contrast-enhanced staging CT scans and follow-ups covered the thorax and the abdomen including the pelvis. According to RECIST 1.1, measurable and non-measurable target lesions were defined at baseline to calculate the sum of longest diameters (SLD). At every follow-up (follow-up time as described above), potential new tumor lesions were evaluated and all previous lesions were reassessed to semiautomatically calculate the SLD and determine the overall response: Complete response (CR; disappearance of all target and non-target lesions), partial response (PR, ≥30% decrease of the SLD compared to baseline), stable disease (SD; no sufficient change compared with baseline or nadir (lowest observed tumor burden)), or progressive disease (PD; ≥20% increase compared with nadir or detection of unequivocal new lesions).

### 2.6 Statistical analysis

Data was analyzed using Prism version 9.2 (Graphpad Software Inc., San Diego, USA). All tests were performed as indicated and resulting p-values were displayed (**P<0.01).

## 3 Results

**Figure 1:**
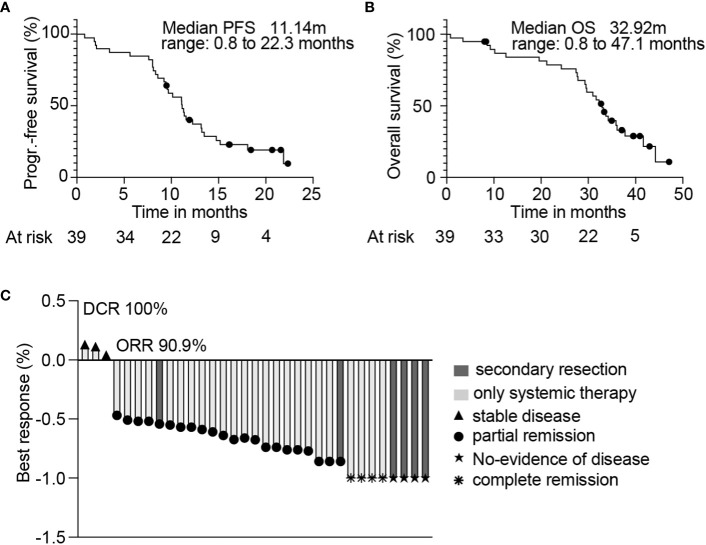
Kaplan-Meier estimates of 39 patients from the AVETUX trial for PFS **(A)** and OS **(B)**. Number of patients at specific timepoints are indicated. Median PFS or OS is depicted. **(C)** Best response achieved and confirmed in radiological review is presented. Secondary resection, chemotherapy only, stable disease, partial remission, no evidence of disease (after resection) or complete remission is indicated.

Forty-three patients were enrolled in the AVETUX trial between June 2017 and July 2018. After central tissue review, four patients were excluded from the data analysis due to RAS (n=3) or BRAF (n=1) mutations, and 39 patients were finally evaluated for response. The median age of these patients was 62 years (inter quartile range (IQR): 51 – 71 years) and most patients had left sided tumors (92%) and liver metastases (77%). The median follow-up was 33 months and the median number of cycles administered was 18 cycles (8 cycles of oxaliplatin, 12 of cetuximab, 13 of 5-FU and 16 of avelumab). The median time of treatment with both avelumab and cetuximab was 5.4 months. The final PFS was 11.1 months (range: 0.8 to 22.3 months; **Figure 1A**), and OS was 32.9 months (range: 0.8 to 47.1 months; **Figure 1B**).

### 3.1 Central computed tomography review

**Figure 2:**
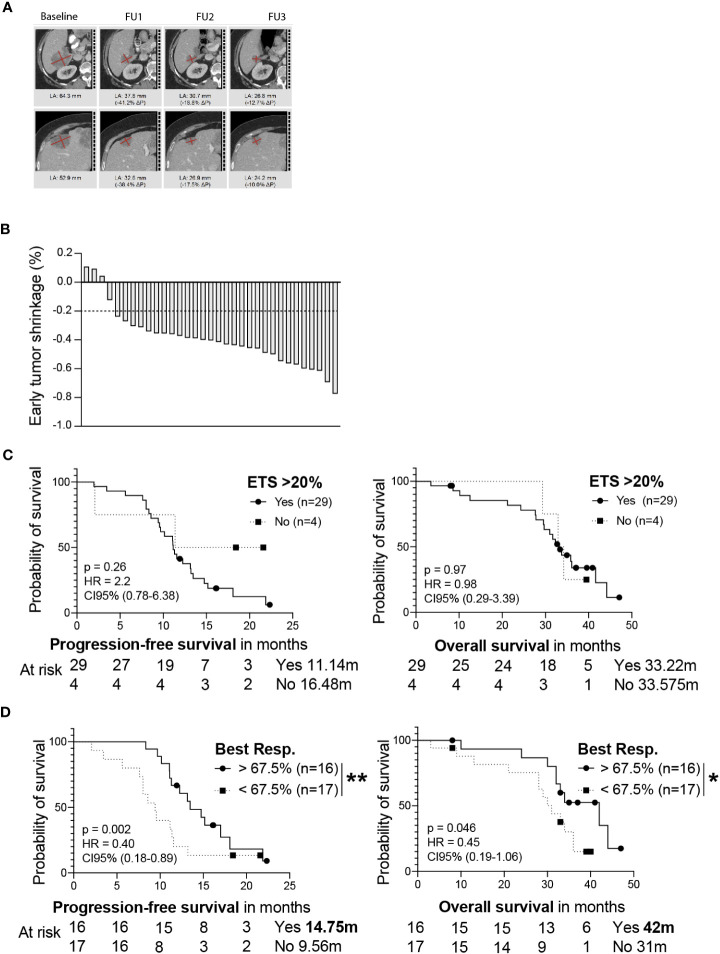
**(A)** Central radiological review of CTs from patient 49002-011 are depicted as an example. Target lesions are shown at baseline and FU examination 1 to 3. **(B)** Percentage of the patient’s early tumor shrinkage was calculated as the difference of the first follow-up CT scan relative to the baseline CT scan. A 20% cut off is depicted. PFS (left) and OS (right) is shown for patients either experiencing or not ETS of at least 20% **(C)**. PFS (left) or OS (right) for patients experiencing a depth of response greater than 67.5% tumor shrinkage or patients experiencing a worse response is shown **(D)**. Median survival of each group and patients at risk are depicted. Statistical significance was calculated using a log-rank Mantel-cox test, * indicates a p value of < 0.05, ** indicates a p value <0.01.

Next, we wondered whether subgroups of patients with distinct response patterns benefitted particularly from the treatment. To address this, we collected computed tomography scans (CTs) from patients in the AVETUX cohort and performed a central radiological review to define the patient’s individual response. Of the 39 AVETUX patients, CTs from 33 patients (85%) were available for analysis. The main characteristics of the herein analyzed cohort was not different from the original AVETUX cohort (**Table 1**). A median of 7 CTs (range 2-8) per patient and a total of 198 CTs were reviewed. We observed 4 patients with complete remissions and 4 others with no-evidence of disease (NED) after secondary resection (CR + NED, 24.2%). Furthermore, 22 partial remissions (PR, 66.7%) and 3 disease stabilizations (SD, 9.1%) indicate an overall strong response to treatment (**Figure 1C**). Next, we analyzed whether early tumor shrinkage (ETS) predicts PFS and OS, as previously reported for anti-EGFR based treatment (18). For this purpose, we divided the cohort into patients with tumor shrinkage of at least 20% or less than 20% (median) tumor shrinkage at the first follow-up CT scan (**Figures 2A, B**). The first follow-up CT scan was performed at a median of 72 days after the baseline CT scan (53-119 days). A ETS of at least 20% was observed in 29 (88%) patients. However, ETS did not indicate better PFS or OS (**Figure 2C**). Given the strong overall response to this treatment regimen, we wondered whether the depth of response was indicative of better PFS and OS. We calculated the depth of response as the maximum tumor shrinkage compared with baseline tumor mass using RECIST 1.1 criteria, as previously reported (19). Of note, the best response was reached after a median of 33 (10-53) weeks and patients who achieved a depth of response to treatment above the median showed significantly higher PFS and OS compared with those below the median (**Figure 2D**). The observed PFS was 14.8 compared to 9.6 months (ratio: 1.54, 95%CI: 0.72 to 3.30; p= 0.0112; HR: 0.40, 95%CI: 0.18 to 0.89) and OS was 42 compared to 31 months (ratio: 1.36, 95%CI: 0.58 to 3.2; p= 0.046; HR: 0.45, 95%CI: 0.19 to 1.10), respectively.

**Table 1:** Patient characteristics of the response evaluated AVETUX cohort and the central review cohort.

Baseline Characteristics		
	Total AVETUX cohort	Central review cohort
Sex, n (%)		
Male	26 (66.7)	22 (66.7)
Female	13 (33.2)	11 (33.2)
Age		
Median years (range)	62 (29-82)	61 (29-74)
Microsattelite status, n (%)		
MSI-H	2 (5)	2 (6)
MSI-L	1 (3)	1 (3)
MSS	36 (92)	30 (91)
Prior adjuvant therapy, n (%)		
Oxaliplatin based	9 (23)	8 (24)
Metastatic sites, n (%)		
Liver	30 (77)	26 (79)
Liver only	13 (33)	10 (30)
Lung	12 (31)	11 (33)
Lymph node	18 (46)	18 (55)
Primary site, n (%)		
Right	3 (8)	2 (6)
Left	36 (92)	31 (94)
Surgery on primary tumor, n (%)	31 (79)	25 (75)
More than 1 metastatic site, n (%)	19 (49)	16 (48)

### 3.2 Evaluation of response biomarkers

**Figure 3:**
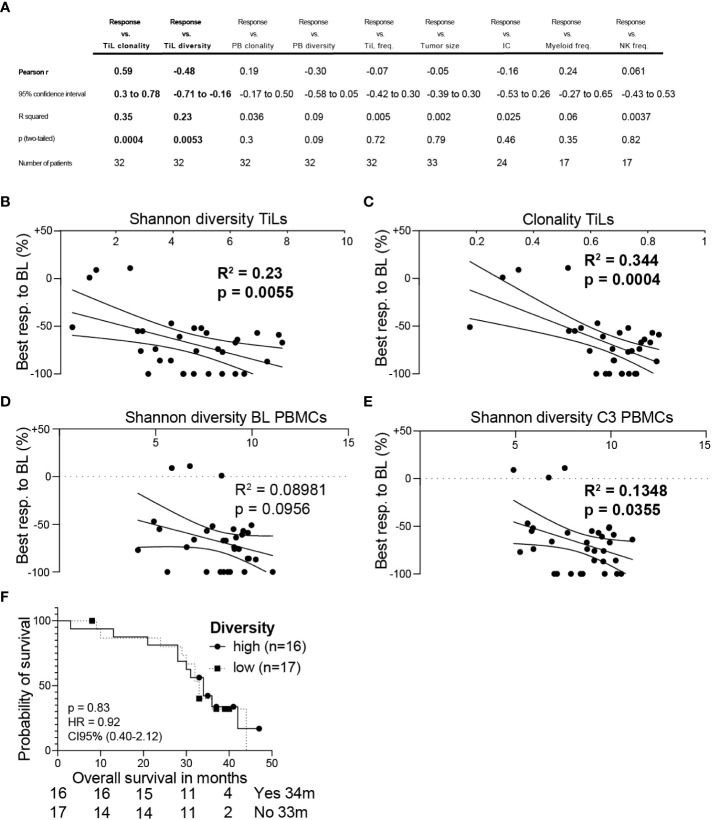
**(A)** Multi linear correlation analysis of indicated markers with the best radiological response. Shannon diversity of TiLs **(B)** or clonality of TiLs **(C)** was correlated with radiological response. Shannon diversity of baseline (BL) PBMCs **(D)** or PBMCs after three cycles of chemotherapy **(E)** was correlated with radiological response. **(F)** The analyzed cohort was separated into patients having a higher than median TiL diversity vs. lower TiL diversity. Overall survival is shown in the Kaplan-Meier estimator. Multi linear correlation and Pearsons´s r **(A)**, simple linear regression **(B–E)** or log-rank Mantel-cox test **(F)** was calculated. P values are indicated.

To define new biomarkers to predict treatment response to the AVETUX regimen, we correlated factors that can predict the response to ICB in other cancers with the depth of response observed in the AVETUX trial cohort. We selected markers available from the translational program of the AVETUX trial like Tumor infiltrating lymphocyte (TiL), myeloid cell and NK cell frequencies; tumor mass calculated at baseline in CT scans; TiL and peripheral blood mononuclear cell (PBMC) clonality and diversity and PD-L1 expression measured as IC score. In a multi linear correlation analysis, the only factors that correlated with response to treatment were TiL diversity and clonality (clonality: R^2^ = 0.35, p= 0.0004; diversity: R^2^ = 0.23, p= 0.0053; **Figures 3A–C**). Considering that PBMCs are more accessible and a source of anti-tumor reactive T cells that overlap, at least in part, with TiLs and change according to the response to ICB in melanoma patients (20), we wondered whether PBMC diversity changes during treatment and correlates with depth of response. We observed that the diversity of PBMCs correlated with response after three cycles of chemotherapy (R^2^ = 0.14, p= 0.036), but not before the initiation of treatment (R^2^ = 0.09, p= 0.096), indicating a change in diversity upon treatment in responder patients (**Figures 3D, E**). Although we found a strong overall correlation of T cell diversity with radiological response, we did not detect an OS benefit for patients with more diverse TiLs compared to patients with lower diversity (**Figure 3F**).

## 4 Discussion

Immunotherapies have revolutionized the treatment of many solid cancers. However, most mCRC patients harbor MSS tumors and do not benefit from classical immunotherapies (21). The AVETUX trial tested the combination of targeted antibodies, chemotherapy, and ICB, among others, to revoke the immunogenicity of MSS mCRC. We here report the final overall survival, central radiological review, and correlate the patient response with potential biomarkers for combinatorial immunotherapy treatments in MSS mCRC.

We did observe a median overall survival of 32.9 months, which is in the lower range of current first-line EGFR antibody and FOLFOX regimen selected not only for KRAS but for all RAS and BRAF status and sidedness (22, 23). We also observed a very high depth of response with a median tumor shrinkage of 67.5%. Of note, previous reports of first-line regimen with a chemotherapy doublet and cetuximab showed a depth of response of only 49% (19), potentially indicating increased efficacy due to the additional ICB. However, a limitation of our study is that we were not able to retrieve CT scans from all recruited patients, overall study a small cohort of patients and performed not pre-defined analysis. Specifically, data were available from only 33 of the 36 included MSS and BRAF/RAS wildtype patients. Nevertheless, the patient characteristics of the cohort analyzed herein are comparable to those of the whole AVETUX trial cohort. In contrast to other studies, we did not detect a role of ETS in distinguishing patients with better or worse PFS and OS. One possible explanation for this observation is the high overall response rate that was achieved only at a median of 33 weeks of treatment. An alternative explanation might be the diversity of metastatic sites and tumor burden in this relatively small cohort of 33 patients. In contrast, the median depth of response allowed the cohort to be subdivided into patients with better or worse PFS and OS.

Similarly, another recent trial found that the addition of the ICB atezolizumab to FOLFOXIRI and bevacizumab increased response in mCRC patients (24). The observed PFS rates were 13.1 months in the atezolizumab arm compared to only 11.5 month in the control arm (p=0.012), and the overall response rate was 81%. In total, only 2 MSI-H patients were included, which is why these data likely reflect the response in the pMMR group. Therefore, ICB in combination with chemotherapy and antiangiogenic or anti-EGFR treatment may be effective in mCRC patients. However, not all patients respond to these regimens, and biomarkers predicting response to these novel combinatorial treatments are lacking. Identification of these markers may allow patient stratification to reduce toxicity, costs, and potentially allocate non-responder patients to other treatment approaches. In addition, the applicability of new and complex treatment regimens, as observed for e.g., FOLFOXIRI and VEGF- or anti-EGFR-antibodies (25), may be hampered in mCRC due to the high response rates of the backbone chemotherapy alone and response predicting markers may allow clinical stratification of patients. If asked to speculate, this may be especially important for patients who are in need of maximal response to treatment to achieve a potential secondary resection.

Most predictors for treatment response to ICB are established in ICB only treatment settings, and markers to assess the response to chemotherapy and immunotherapy combinations are less well established in all cancers and missing in mCRC (26, 27). To address this, we correlated known ICB single-agent response markers from other cancers, such as TiL frequency, diversity, PD-L1 expression measured as the IC score, tumor mass, and myeloid and NK cell infiltration, with depth of response to treatment. Among these, we only found TiL clonality and diversity to correlate with depth of response. Moreover, PBMC diversity correlated with depth of response after three cycles of chemotherapy, but not at baseline. Both TiL and PBMC diversity after treatment initiation are prognostic in ICB treated melanoma patients, and TiL diversity is also prognostic in a range of other cancers (20, 28). These data support a potential role for TiLs in indicating response to the AVETUX regimen, however, both TiL diversity and clonality did not separate the cohort into patients with better or worse OS. One explanation for this might be that the anti PD-L1 antibody avelumab leads to an early anti-tumor immune activation, thereby inducing high response rates, but this effect may be lost in the course of treatment e.g., through the emergence of immune escape variants as suggested earlier (14). An alternative hypothesis is that due to the small patient cohort, the high overall response rate, the different tumor sizes, and the availability of second- and third-line therapies, other factors are strongly contributing to OS in addition to the first-line treatment response.

We would like to highlight that the tested treatment was feasible in terms of toxicity which was measured as adverse events and was in the range of individual treatment with FOLFOX/anti-EGFR and ICB (14).

In summary, TiL diversity and clonality, as well as PBMC diversity after three cycles of chemotherapy may be potential markers for treatment response to ICB chemotherapy combinations in mCRC. These findings need to be validated in a larger cohort, and the predictive power of these markers need to be evaluated. If successful, such a marker may be used to stratify patients or design clinical trials to increase the number of patients benefitting from ICB in MSS mCRC.

## Data availability statement

The datasets presented in this study can be found in online repositories. The names of the repository/repositories and accession number(s) can be found in the article/supplementary material.

## Ethics statement

The studies involving human participants were reviewed and approved by Ethik-Kommission der Ärztekammer Hamburg. The patients/participants provided their written informed consent to participate in this study. Written informed consent was obtained from the individual(s) for the publication of any potentially identifiable images or data included in this article.

## Author contributions

Conceptualization: JT, AS, EG, and MBi; methodology: JT, IR, MS, AS, DS, CS, LvW, LP, RS, MBa, CM, BS, CW, and MBi; investigation: JT, AS, DS, CS, LvW, LP, RS, MBa, CM, BS, CW, MBi, EG, and SH-B; formal analysis: EW and AH; data curation: JT, IR, and MS; writing – original draft: JT and AS; writing – review and editing: JT, IR, MS, AS, LvW, DS, and CB; recources: JT, IR, MS, MBi, SL, JR-K, RD, TE, SD, S-EA-B, MK, UP, CB, AS, EG, and SH-B; supervision: JT, MBi, and AS. All authors had access to the study data and reviewed and approved the final manuscript
